# Estimated Glucose Disposal Rate: A Potential Determinant for Microvascular and Macrovascular Complications in Type 2 Diabetes

**DOI:** 10.1002/edm2.70037

**Published:** 2025-03-23

**Authors:** Ehsan Bahrami Hezaveh, Rana Hashemi, Mohammadamin Noorafrooz, Fatemeh Mohammadi, Amirhossein Yadegar, Sahar Karimpour Reyhan, Manouchehr Nakhjavani, Alireza Esteghamati, Soghra Rabizadeh

**Affiliations:** ^1^ Endocrinology and Metabolism Research Center (EMRC), Vali‐Asr Hospital, Imam Khomeini Hospital Complex (IKHC) Tehran University of Medical Sciences Tehran Iran

**Keywords:** coronary artery disease, estimated glucose disposal rate, insulin resistance, macrovascular complications, microvascular complications, nephropathy, retinopathy, type 2 diabetes

## Abstract

**Objective:**

This study investigates the association between estimated glucose disposal rate (eGDR), a measurement of insulin resistance, and microvascular and macrovascular complications in patients with type 2 diabetes (T2D).

**Methods:**

This cross‐sectional study enrolled 7471 patients with T2D from 2010 to 2023. The eGDR was calculated using waist circumference, HbA1C levels, and hypertension status. Logistic regression analysis and restricted cubic splines were utilised to examine the relationship between eGDR and vascular complications, including nephropathy, retinopathy, and coronary artery disease (CAD). The robustness of the results and between‐group interactions were examined by sensitivity and subgroup analysis. Furthermore, receiver operating characteristic (ROC) curve analysis was employed to assess the discriminatory value of the adjusted model for T2D vascular complications.

**Results:**

Among participants, 56.5% were female, with a mean age of 57.04 ± 11.05 years and a median of 8 years of diabetes duration. In the final adjusted model, each unit increase in the standard deviation of eGDR was significantly associated with a 23.6%, 24.8% and 29.6% decrease in the odds of nephropathy, retinopathy, and CAD, respectively. There was a significant association between higher eGDR quartiles compared to Q1 for all complications (*p* < 0.05). The Q4 group had the lowest adjusted odds ratios (ORs) compared to the Q1 group for all complications; the OR of Q4 was 0.549 for nephropathy, 0.360 for retinopathy, and 0.396 for CAD (*p* < 0.001). The restricted cubic spline for nephropathy followed a negative nonlinear association with eGDR, whereas for retinopathy and CAD, it followed a negative linear pattern. The effect of eGDR was consistent among different subgroups. The ROC curve analysis of the adjusted model showed good discriminatory power for all complications.

**Conclusion:**

In patients with type 2 diabetes, a higher eGDR was significantly associated with a lower risk of microvascular and macrovascular complications, regardless of well‐known confounders.

## Graphical abstract

In patients with type 2 diabetes, a higher eGDR (an indicator of insulin resistance) is significantly associated with a lower risk of microvascular and macrovascular complications, including nephropathy, retinopathy, and coronary artery disease (CAD).

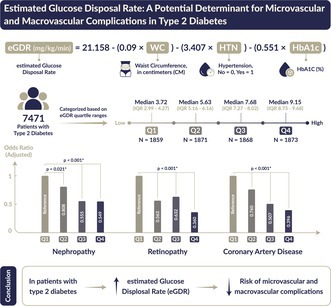

## 1 Introduction

Diabetes is an ongoing global challenge, with an increasing prevalence affecting millions of people worldwide and placing a considerable burden on individuals and healthcare systems [1, 2]. Despite the breakthroughs in managing the disease progression, many patients with type 2 diabetes (T2D) still develop life‐threatening complications [3]. Preventing these adverse events requires multidimensional interventions, many of which may be invasive and costly for patients and healthcare systems.

Haemoglobin A1c (HbA1c) levels remain a crucial indicator for predicting and preventing vascular complications of diabetes [4, 5]. However, despite achieving controlled HbA1c levels through intensive hyperglycaemia therapy, a considerable proportion of patients still experience vascular complications [6, 7]. Microvascular complications of T2D significantly contribute to morbidity and daily life consequences for patients, while the majority of T2D patient mortality can be attributed to macrovascular complications [8, 9, 10]. Preventing and managing microvascular complications as a predictor of macrovascular complications can significantly lower serious cardiovascular events and premature deaths in patients with T2D [11].

The complex nature of disease pathology and clinical progression mandates developing and utilising effective indicators to assess disease course. Several investigations have revealed the key role of insulin resistance in developing diabetes complications [12, 13, 14, 15]. Emerging evidence indicates that insulin resistance not only dysregulates glycemic metabolism but also contributes to oxidative stress, inflammation, and coagulation, potentially leading to complications in patients with T2D [16, 17, 18].

The euglycemic hyperinsulinemic clamp technique is the gold standard for measuring insulin resistance. However, this approach's high cost and invasive nature make it impractical for implementation in large clinical settings. The estimated glucose disposal rate (eGDR), which is determined using readily available clinical parameters, was developed for the estimation of insulin resistance in diabetes patients. The eGDR, a valid insulin resistance surrogate, has shown a strong correlation with the glucose disposal rate determined by a euglycemic hyperinsulinemic clamp. It is considered superior to the homeostasis model assessment of insulin resistance (HOMA‐IR) in patients with type 2 diabetes [13]. The eGDR formula's components can be obtained by routine clinical and paraclinical measurements, making it a practical noninvasive approach for the assessment of insulin resistance in patients with diabetes.

Although previous studies have examined the relationships between insulin resistance (assessed by the eGDR) and type 1 diabetes (T1D) complications and outcomes [19, 20, 21, 22, 23], the associations between eGDR and T2D complications have not been sufficiently studied. Moreover, some studies have explored the association between the eGDR and individual complications in patients with T2D. Still, limited large‐scale studies have investigated the association between the eGDR and both microvascular and macrovascular complications together.

The present study explored the associations between insulin resistance assessed by the eGDR and microvascular and macrovascular complications of type 2 diabetes (T2D), including nephropathy, retinopathy, and coronary artery disease (CAD).

## 2 Materials and Methods

### 2.1 Study Design

This study included 7471 patients with type 2 diabetes (T2D) after excluding 691 patients due to incomplete or missing information. Participants' demographic and clinical data was collected from an ongoing cohort study at Vali‐Asr Hospital, affiliated with Tehran University of Medical Sciences, and individuals admitted to the endocrinology and diabetes clinic in Vali‐Asr Hospital between 2010 and 2023 were included. Inclusion criteria were: confirmed diagnosis of T2D and age 18 years or more at the time of enrollment. The diagnosis of T2D was based on the American Diabetes Association (ADA) diagnostic criteria [24]. Exclusion criteria were pregnancy, incomplete or missing data for the calculation of eGDR, and missing data on the complications of nephropathy, retinopathy, or CAD. Each participant signed an informed consent form before participating in the study. The study design was approved by the Ethics Committee of Tehran University of Medical Sciences.

### 2.2 Data Collection

The demographic characteristics and complications of all participants with T2D, including age, sex, duration of diabetes, smoking status, waist circumference, body weight, height, body mass index (BMI), and medication history, were collected. Clinical parameters and laboratory test results, including systolic and diastolic blood pressure (SBP and DBP), total cholesterol, high‐density lipoprotein cholesterol (HDL‐C), low‐density lipoprotein cholesterol (LDL‐C), triglyceride (TG), aspartate aminotransferase (AST), alanine aminotransferase (ALT), blood urea nitrogen (BUN), estimated glomerular filtration rate (eGFR), serum creatinine, glycated haemoglobin (HbA1c), and fasting blood glucose, were collected. The Chronic Kidney Disease Epidemiology Collaboration (CKD‐EPI) creatinine formula (2021) was used for calculating the eGFR [25]. Blood pressure was measured using an Omron M7 sphygmomanometer (Hoofddorp, The Netherlands) while the patient was seated and after 10 min of rest. A second measurement was conducted 15 min later, and the mean of the two measurements was recorded. The following equation was used for BMI: body weight (kg) divided by squared height (m^2^).

The following equation was used for calculating the eGDR (mg/kg/min): eGDR = 21.158 − (0.09 × waist circumference) − (3.407 × hypertension) − (0.551× HbA1c); waist circumference is in centimetres (cm), hypertension (no = 0, yes = 1), and HbA1c = HbA1c (%) [26]. Patients were classified as hypertensive if they met one or more of the following criteria: systolic blood pressure higher than 140 mmHg, diastolic blood pressure higher than 90 mmHg, or using antihypertensive medication(s).

### 2.3 Study Outcomes

The main outcomes were microvascular and macrovascular complications of T2D, including retinopathy, nephropathy and CAD. All microvascular and macrovascular complications were defined based on ADA diagnostic criteria [27, 28]. Retinopathy was defined as a fundoscopic change in the retina diagnosed by an ophthalmologist. The diagnosis of nephropathy was based on the presence of ongoing albuminuria, which was defined as a urinary albumin‐to‐creatinine ratio (UACR) equal to or greater than 30 mg/g or an estimated glomerular filtration rate (eGFR) less than 60 mL/min/1.73 m^2^. CAD was defined based on a prior history of one or more of the following: acute coronary syndrome, coronary artery bypass graft CABG, or percutaneous coronary intervention (PCI).

### 2.4 Statistical Analysis

The participants were categorised based on their eGDR quartile ranges (Q1–Q4). Continuous variables were expressed as the mean ± standard deviation (SD) for normally distributed variables or as the median with interquartile range (IQR) for variables with a skewed distribution. Based on the distribution of variables, comparisons between the groups were conducted by employing one‐way analysis of variance (ANOVA) or the Kruskal–Wallis test. The categorical variables were expressed as numbers (percentages) and analysed using the *χ* ^2^ test.

The associations between the eGDR as a continuous variable or eGDR quartiles as categorical variables and the microvascular and macrovascular complications of diabetes (retinopathy, nephropathy, and CAD) were investigated using three logistic regression models for each complication. The first eGDR quartile was used as the reference in the regression. Model 1 was not adjusted for any confounders. Model 2 was adjusted for the age and sex of the participants. Model 3 was adjusted for confounders mentioned in Model 2, plus duration of diabetes, BMI, smoking status, eGFR, HDL‐C, LDL‐C and TG. However, WC, hypertension, antihyperglycemic drugs, antihypertensive drugs and HbA1c were not included in the final regression model, given that these variables are included in the eGDR formula and are related to the formula components. Additionally, lipid‐lowering drug use was not included in the adjusted model because of the inclusion of lipid parameters. In all models, the variance Inflation Factor (VIF) was used for screening collinearity with the cut‐off of VIF < 2.5 [29]. A restricted cubic spline was utilised for the final adjusted model to evaluate the dose–response association of eGDR with nephropathy, retinopathy and CAD. Furthermore, sensitivity and subgroup analyses were performed to examine the robustness of our results and evaluate the potential between subgroup differences. As a sensitivity analysis, we excluded outliers from the data based on the values of eGDR (*z* > |3|) and performed logistic regression analysis for all complications again. The associations were reported with *p*‐values and odds ratios (ORs) with 95% confidence intervals (CIs). Two‐sided *p*‐values of less than 5% were considered to indicate statistical significance.

Receiver operating characteristic (ROC) curves were generated to assess the discriminatory value of the adjusted model for microvascular and macrovascular complications of T2D. The area under the curve (AUC) was calculated, and the optimal cutoff values for eGDR with specificity and sensitivity were established. All data were analysed using R software version 4.4.1, SPSS version 27, and GraphPad Prism 9.

## 3 Results

### 3.1 Baseline Characteristics

**FIGURE 1:**
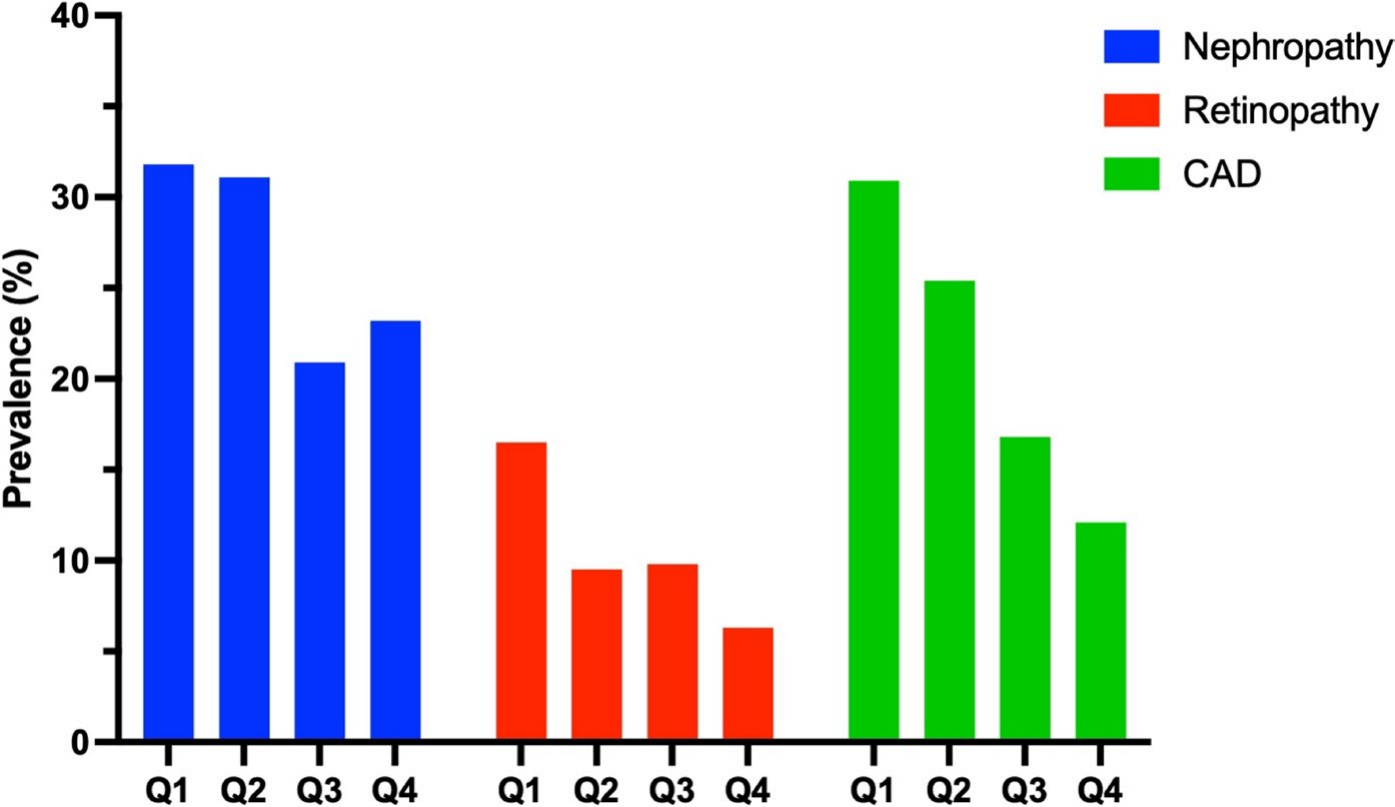
Prevalence of vascular complications across eGDR quartiles (colour online only).

The present study included 7471 patients with T2D. Among the participants, 56.5% were female, and the mean age was 57.04 ± 11.05 years, with a median diabetes duration of 8 years (IQR: 3–14). The median HbA1c was 7.60 (6.60–8.90), and the median BMI was 28.35 (25.63–31.74) among all participants. The demographic, clinical, and laboratory characteristics of the patients based on eGDR quartiles are presented in Table 1. Age, duration of diabetes, weight, WC, HC, SBP, FBS, HbA1c, TG, serum creatinine, AST, ALT, GFR and BMI were significantly lower in the Q4 group than in the other three quartiles. However, HDL and LDL were significantly greater in the Q4 subgroup than in the other quartiles. The proportions of patients with CAD, nephropathy, and retinopathy were highest in the Q1 quartile (Figure 1). The median eGDRs for Q1–Q4 were 3.72 (IQR: 2.99–427), 5.63 (IQR: 5.16–6.16), 7.68 (IQR: 7.27–8.02), and 9.15 (IQR: 8.75–9.68), respectively.

**TABLE 1:** Baseline characteristics of patients with type 2 diabetes categorised into quartiles of estimated glucose disposal rate (eGDR).

Variables	All patients (*N* = 7471)	Q1: eGDR ≤ 4.74 (*N* = 1859)	Q2: 4.74 < eGDR ≤ 6.74 (*N* = 1871)	Q3: 6.74 < eGDR ≤ 8.38 (*N* = 1868)	Q4: eGDR > 8.38 (*N* = 1873)	*p* for Q1–Q4
Age	57.04 ± 11.05	59.19 ± 10.16	58.42 ± 11.07	55.61 ± 11.02	54.97 ± 11.31	< 0.001
Sex (female); *n* (%)	4221 (56.5)	1177 (61.7)	1053 (56.3)	951 (50.9)	1070 (57.1)	< 0.001
Duration of diabetes (years)	8 (3–14)	10 (5–16)	8 (4–14)	7 (3–13)	6.0 (3–11)	< 0.001
Height (cm)	162 (155–170)	160 (155–168)	161 (155–169)	163 (157–171)	162 (155–170)	< 0.001
Weight (kg)	75.0 (67.0–85.0)	81 (73–90)	74 (66–84)	77 (69–85)	69 (63–76)	< 0.001
Waist circumference (cm)	98.0 (91.0–104.0)	104 (98–111)	97 (91–104)	100 (93–105)	91 (85–96)	< 0.001
Hip circumference (cm)	104.0 (100.0–110.0)	109 (103–115)	105 (100–110)	105 (101–110)	101 (97–104.5)	< 0.001
Smoking status; *n* (%)	381 (5.1)	111 (6.0)	93 (5.0)	95 (5.1)	82 (4.4)	0.172
SBP (mmHg)	130.0 (120.0–140.0)	140 (129–150)	130 (120–142)	125 (118–135)	120 (110–130)	< 0.001
DBP (mmHg)	80.0 (72.0–82.0)	80 (79–90)	80.0 (72–84)	80 (70–80)	80 (70–80)	< 0.001
FBS (mg/dL)	150 (123–196)	170 (137–217)	150 (123–201)	157 (128–203)	132 (114–160)	< 0.001
HbA1C (%)	7.60 (6.60–8.90)	8.5 (7.6–9.8)	7.4 (6.5–9.0)	8.0 (7.0–9.0)	6.7 (6.0–7.4)	< 0.001
Total cholesterol (mg/dL)	175 (147–207)	174 (148–207)	169 (142–202)	180 (149–210)	177 (150–206)	< 0.001
HDL (mg/dL)	43 (37–51)	43 (37–50)	44 (37–51)	42 (36–51)	45 (38–52)	< 0.001
LDL (mg/dL)	97 (76–123)	97 (76–121)	93 (72–120)	98 (77–126)	99 (78–124)	< 0.001
TG (mg/dL)	150 (108–212)	163 (118–225)	150 (112–210)	155 (108–221)	134 (98–189)	< 0.001
Creatinine (mg/dL)	1.0 (0.8–1.1)	1.00 (0.88–1.14)	1.00 (0.83–1.10)	1.00 (0.80–1.10)	0.96 (0.80–1.10)	< 0.001
AST (mg/dL)	20 (16–25)	20 (16–25)	20 (16–26)	20 (16–26)	19 (15–24)	0.008
ALT (mg/dL)	22 (17–31)	22 (17–32)	22 (17–30)	23 (17–32)	21 (16–29)	< 0.001
eGFR (ml/min 1.73 m2)	78.36 (65.70–91.63)	74.49 (61.44–88.04)	77.75 (63.69–90.83)	80.96 (69.61–94.28)	80.39 (68.63–92.57)	< 0.001
BMI (kg/mg^2^)	28.35 (25.63–31.74)	31.04 (28.04–34.80)	28.32 (25.71–31.64)	28.57 (26.02–31.56)	26.06 (23.87–28.54)	< 0.001
Hypertension; *n* (%)	3160 (42.3)	1806 (97.1)	1255 (67.1)	98 (5.2)	1 (0.1)	< 0.001
CAD; *n* (%)	1588 (21.3)	574 (30.9)	475 (25.4)	313 (16.8)	226 (12.1)	< 0.001
Nephropathy; *n* (%)	1998 (26.7)	591 (31.8)	582 (31.1)	391 (20.9)	434 (23.2)	< 0.001
Retinopathy; *n* (%)	674 (10.4)	253 (16.5)	154 (9.5)	162 (9.8)	105 (6.3)	< 0.001
Neuropathy; *n* (%)	1015 (24.3)	310 (29.6)	250 (23.7)	265 (25.2)	190 (18.7)	< 0.001
Using HTN medication; *n* (%)	3100 (41.5)	1681 (90.4)	1201 (64.2)	152 (8.1)	66 (3.5)	< 0.001
Using lipid lowering medication; *n* (%)	5616 (75.2)	1486 (79.9)	1451 (77.6)	1363 (73.0)	1316 (70.3)	< 0.001
DM medication; *n* (%)						0.075
Oral	7398 (99.0)	1847 (99.4)	1849 (98.8)	1845 (98.8)	1857 (99.1)	
Oral + Insulin	34 (0.5)	7 (0.4)	14 (0.7)	7 (0.4)	6 (0.3)	
Insulin	39 (0.5)	5 (0.3)	8 (0.4)	16 (0.9)	10 (0.5)	
eGDR (mg/kg/min)	6.37 (4.73–8.38)	3.72 (2.99–4.27)	5.63 (5.16–6.16)	7.68 (7.27–8.02)	9.15 (8.75–9.68)	< 0.001

Abbreviations: ALT, alanine aminotransferase; AST, aspartate aminotransferase; BMI, body mass index; BUN, blood urea nitrogen; CAD, coronary artery disease; DBP, diastolic blood pressure; DM, diabetes mellitus; eGDR, estimated glucose disposal rate; eGFR, estimated glomerular filtration rate; FBS, fasting blood glucose; HbA1C, haemoglobin A1C; HDL, high‐density lipoprotein; HTN, hypertension; LDL, low‐density lipoprotein; SBP, systolic blood pressure; TG, triglyceride.

### 3.2 Nephropathy

Overall, 26.7% of patients had nephropathy. In Model 1 and Model 2, the ORs of Q3 and Q4 were significantly lower for nephropathy than for the reference quartile (*p* < 0.05). However, the OR of the second quartile was not significantly different from that of the reference quartile in Model 1 or Model 2 (Table 2). There was a significant association between eGDR (as a continuous variable or quartile) and nephropathy in patients after adjusting for confounding variables in Model 3. As a continuous variable, an increased eGDR was significantly associated with a decreased risk of nephropathy in all 3 models. Each SD increase in the eGDR was associated with a 23.6% lower chance of nephropathy after adjusting for confounders in Model 3 (OR = 0.764, 95% CI: 0.710, 0.822; *p* < 0.001). When eGDR was treated as a categorical variable, the ORs for nephropathy were 0.808 (95% CI: 0.674, 0.968) for Q2, 0.555 (95% CI: 0.458, 0.673) for Q3, and 0.549 (95% CI: 0.450, 0.671) for Q4 compared with the reference quartile of eGDR (Q1) after adjustment in Model 3 (Table 2).

**TABLE 2:** Logistic regression analysis with ORs and 95% CIs showing the association of eGDR with nephropathy, retinopathy and CAD.

Complication	Variable	Model 1^a^		Model 2^b^		Model 3^c^	
		OR (95% CI)	*p*	OR (95% CI)	*p*	OR (95% CI)	*p*
Nephropathy	eGDR (per SD)	0.818 (0.777, 0.861)	< 0.001	0.914 (0.864, 0.967)	0.002	0.764 (0.710, 0.822)	< 0.001
	eGDR quartiles						
	Q1	Ref		Ref		Ref	
	Q2	0.969 (0.844, 1.112)	0.652	1.002 (0.863, 1.163)	0.977	0.808 (0.674, 0.968)	0.021
	Q3	0.568 (0.490, 0.659)	< 0.001	0.704 (0.600, 0.826)	< 0.001	0.555 (0.458, 0.673)	< 0.001
	Q4	0.647 (0.560, 0.748)	< 0.001	0.853 (0.729,0.998)	0.046	0.549 (0.450, 0.671)	< 0.001
	*p*_trend_ for Q1–Q4		< 0.001		< 0.001		< 0.001
Retinopathy	eGDR (per SD)	0.719 (0.664, 0.779)	< 0.001	0.753 (0.694, 0.817)	< 0.001	0.752 (0.682, 0.830)	< 0.001
	eGDR quartiles						
	Q1	Ref		Ref		Ref	
	Q2	0.533 (0.431, 0.660)	< 0.001	0.545 (0.440, 0.676)	< 0.001	0.563 (0.444, 0.712)	< 0.001
	Q3	0.547 (0.443, 0.676)	< 0.001	0.610 (0.493, 0.756)	< 0.001	0.632 (0.499, 0.801)	< 0.001
	Q4	0.337 (0.266, 0.428)	< 0.001	0.377 (0.296, 0.481)	< 0.001	0.360 (0.271, 0.477)	< 0.001
	*p*_trend_ for Q1–Q4		< 0.001		< 0.001		< 0.001
CAD	eGDR (per SD)	0.645 (0.609, 0.682)	< 0.001	0.664 (0.625, 0.705)	< 0.001	0.704 (0.659, 0.752)	< 0.001
	eGDR quartiles						
	Q1	Ref		Ref		Ref	
	Q2	0.762 (0.660, 0.879)	< 0.001	0.739 (0.636, 0.859)	< 0.001	0.760 (0.646, 0.895)	< 0.001
	Q3	0.451 (0.385, 0.527)	< 0.001	0.474 (0.402, 0.559)	< 0.001	0.507 (0.424, 0.605)	< 0.001
	Q4	0.307 (0.259, 0.364)	< 0.001	0.335 (0.281, 0.400)	< 0.001	0.396 (0.326, 0.479)	< 0.001
	*p*_trend_ for Q1–Q4		< 0.001		< 0.001		< 0.001

Abbreviations: CI, confidence interval; eGDR, estimated glucose disposal rate; OR, odds ratio; SD, standard deviation.

^a^ Not adjusted for any confounders.

^b^ Adjusted for age and sex.

^c^ Adjusted for age, sex, duration of diabetes, BMI, smoking status, eGFR, HDL‐C, LDL‐C, and TG.

### 3.3 Retinopathy

Retinopathy was observed in 10.4% of patients. According to Table 2, the ORs for retinopathy were significant in all three models, regardless of whether the eGDR was treated as a continuous or categorical variable (quartiles) (*p* < 0.001). After adjusting for confounders in Model 3, there was a 24.8% decrease in the incidence of retinopathy for every SD increase in the eGDR (OR = 0.752, 95% CI: 0.682, 0.830; *p* < 0.001). The crude ORs for retinopathy across different quartiles were significant according to the unadjusted model (OR = 0.533 for Q2, OR = 0.547 for Q3, OR = 0.337 for Q4; *p* < 0.001). After adjusting for several confounding factors in Model 3, the difference remained significant, and the ORs barely changed, varying from 0.360 (95% CI: 0.271, 0.477; *p* < 0.001) for Q4 to 0.632 (95% CI: 0.499, 0.801; *p* < 0.001) for Q3 across different quartiles of the eGDR compared with those for Q1 (Table 2).

### 3.4 Coronary Artery Disease

CAD was present in 21.3% of all patients. According to the unadjusted model for CAD risk, increasing the eGDR as a continuous or categorical variable was significantly associated with a gradual decrease in the risk of CAD. The crude OR of the eGDR (per SD increase) was 0.645 (95% CI: 0.609, 0.682; *p* < 0.001) in the unadjusted model. The OR of Q4 for the eGDR was lowest compared to Q1 according to the unadjusted model (OR = 0.307, 95% CI: 0.259, 0.364; *p* < 0.001). After adjusting for confounding factors, the association between the eGDR and CAD incidence remained significant irrespective of the continuous or categorical nature of the eGDR, revealing the independent risk of the eGDR in Model 2 and Model 3 for CAD. In Model 3, the OR of eGDR (per SD increase) as a continuous variable was 0.704 (95% CI: 0.659, 0.752), showing that for each SD increase in the eGDR, the risk of CAD decreased by 29.6%. The ORs for the eGDR quartiles gradually decreased from Q2 (OR = 0.760, 95% CI = 0.646, 0.895; *p* < 0.001) to Q4 (OR = 0.396, 95% CI = 0.326, 0.479; *p* < 0.001) compared with those of Q1, which was the reference quartile (Table 2).

### 3.5 Restricted Cubic Spline

**FIGURE 2:**
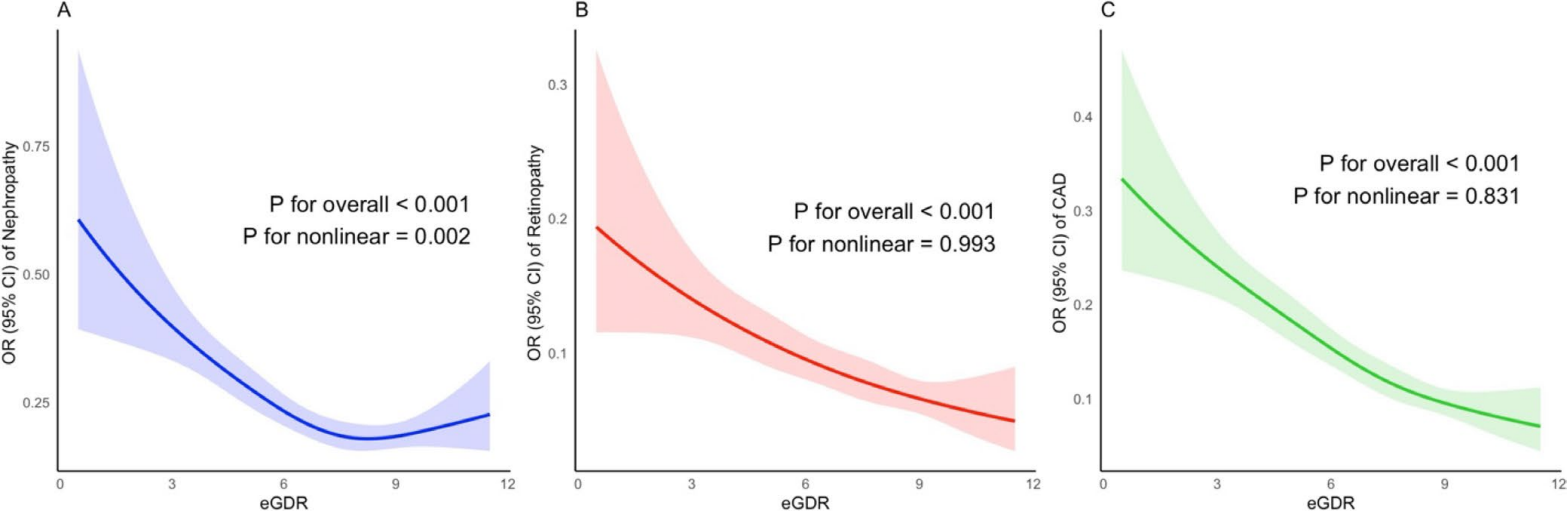
Dose–response relationship between eGDR vascular complications in T2D patients. (A) Nephropathy. (B) Retinopathy. (C) CAD. CAD, coronary artery disease; CI, confidence interval; eGDR, estimated glucose disposal rate; OR, odds ratio; T2D, type 2 diabetes.

The findings of the restricted cubic spline analyses revealed a nonlinear negative correlation between the eGDR and nephropathy (*p* for nonlinearity = 0.002). The odds of nephropathy decreased with increasing eGDR. While nephropathy indicated a sharper decline at lower levels of eGDR, the effect plateaued at higher eGDR levels (Figure 2A). However, the dose–response analyses of eGDR with retinopathy and CAD suggested a linear negative correlation in patients with T2D (*p* for nonlinearity = 0.993 and *p* for nonlinearity = 0.831, respectively), with consistent decreases in retinopathy and the CAD odds ratio as the eGDR increased (Figure 2B,C). These findings suggest that eGDR has a more complex relationship with nephropathy, whereas its associations with retinopathy and CAD can be adequately modelled with a linear term.

### 3.6 Sensitivity Analysis

Similar to the primary analysis, in the majority of the results of the sensitivity analysis, increasing eGDR was associated with a lower risk of nephropathy (Model 3: OR = 0.763, 95% CI: 0.709, 0.822; *p* < 0.001), retinopathy (Model 3: OR = 0.751, 95% CI: 0.680, 0.829; *p* < 0.001), and CAD (Model 3: OR = 0.702, 95% CI: 0.657, 0.749; *p* < 0.001) (Table 3). This finding suggests a consistent negative association between eGDR and both microvascular and macrovascular complications in T2D patients, supporting the robustness of our results.

**TABLE 3:** Logistic regression analysis with ORs and 95% CIs showing the association of eGDR with nephropathy, retinopathy and CAD in sensitivity analysis excluding outlier values of eGDR.

Complication	Variable	Model 1^a^		Model 2^b^		Model 3^c^	
		OR (95% CI)	*p*	OR (95% CI)	*p*	OR (95% CI)	*p*
Nephropathy	eGDR (per SD)	0.816 (0.775, 0.859)	< 0.001	0.913 (0.863, 0.966)	0.001	0.763 (0.709, 0.822)	< 0.001
	eGDR quartiles						
	Q1	Ref		Ref		Ref	
	Q2	0.959 (0.835, 1.101)	0.55	0.993 (0.856, 1.152)	0.931	0.809 (0.675, 0.969)	0.021
	Q3	0.554 (0.478, 0.643)	< 0.001	0.691 (0.589, 0.810)	< 0.001	0.551 (0.454, 0.667)	< 0.001
	Q4	0.658 (0.568, 0.760)	< 0.001	0.869 (0.843, 1.017)	0.081	0.562 (0.460, 0.687)	< 0.001
	*p*_trend_ for Q1–Q4		< 0.001		< 0.001		< 0.001
Retinopathy	eGDR (per SD)	0.717 (0.662, 0.777)	< 0.001	0.752 (0.692, 0.816)	< 0.001	0.751 (0.680, 0.829)	< 0.001
	eGDR quartiles						
	Q1	Ref		Ref		Ref	
	Q2	0.538 (0.434, 0.665)	< 0.001	0.550 (0.443, 0.681)	< 0.001	0.571 (0.451, 0.722)	< 0.001
	Q3	0.538 (0.435, 0.664)	< 0.001	0.603 (0.486, 0.746)	< 0.001	0.627 (0.494, 0.794)	< 0.001
	Q4	0.342 (0.268, 0.433)	< 0.001	0.382 (0.299, 0.486)	< 0.001	0.366 (0.274, 0.485)	< 0.001
	*p*_trend_ for Q1–Q4		< 0.001		< 0.001		< 0.001
CAD	eGDR (per SD)	0.642 (0.607, 0.680)	< 0.001	0.662 (0.623, 0.703)	< 0.001	0.702 (0.657, 0.749)	< 0.001
	eGDR quartiles						
	Q1	Ref		Ref		Ref	
	Q2	0.761 (0.659, 0.879)	< 0.001	0.741 (0.637, 0.860)	< 0.001	0.768 (0.652, 0.903)	0.001
	Q3	0.445 (0.381, 0.520)	< 0.001	0.468 (0.397, 0.551)	< 0.001	0.499 (0.417, 0.596)	< 0.001
	Q4	0.308 (0.259, 0.365)	< 0.001	0.337 (0.282, 0.403)	< 0.001	0.406 (0.334, 0.492)	< 0.001
	*p*_trend_ for Q1–Q4		< 0.001		< 0.001		< 0.001

Abbreviations: CI, confidence interval; eGDR, estimated glucose disposal rate; OR, odds ratio; SD, standard deviation.

^a^ Not adjusted for any confounders.

^b^ Adjusted for age and sex.

^c^ Adjusted for age, sex, duration of diabetes, BMI, smoking status, eGFR, HDL‐C, LDL‐C, and TG.

### 3.7 Subgroup Analysis

Subgroup analysis was carried out to analyse whether the effect of eGDR on complications of T2D varies among different stratifications. Effect modification of eGDR was not observed across any subgroup for all complications, as the interaction analysis yielded non‐significant results (*p* for interaction > 0.05) (Table S1). For the association between nephropathy and eGDR, the analysis revealed significant ORs for males (0.876, 95% CI: 0.794, 0.967), females (0.855, 95% CI: 0.779, 0.938), those younger than 65 years (0.873, 95% CI: 0.804, 0.948), and those 65 years old or older (0.831, 95% CI: 0.733, 0.941). However, the association was significant only in females (OR = 0.868, 95% CI: 0.777, 0.971) and individuals 65 years old or older (OR = 0.828, 95% CI: 0.715, 0.959) for retinopathy. Subgroup analysis also yielded a significant association between eGDR and CAD across all subgroups, including males (OR = 0.756, 95% CI: 0.688, 0.830), females (OR = 0.817, 95% CI: 0.7142 0.899), individuals younger than 65 years old (OR = 0.744, 95% CI: 0.684, 0.809), individuals 65 years old or older (OR = 0.837, 95% CI: 0.752, 0.930), lean (OR = 0.856, 95% CI: 0.743, 0.986), and overweight/obese individuals (OR = 0.767, 95% CI: 0.712, 0.826) (Table S1).

### 3.8 Discriminatory Value of the eGDR

**FIGURE 3:**
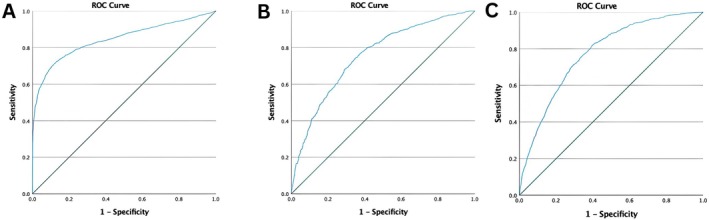
Receiving operating characteristic (ROC) curve analysis of the adjusted model (Model 3) for discriminating vascular complications. (A) Nephropathy, (B) Retinopathy, (C) Coronary Artery Disease (CAD).

ROC curve analysis revealed good discriminatory power of the adjusted model for predicting nephropathy, retinopathy, and CAD. The AUC of the adjusted model for distinguishing between patients with and without complications was 0.845 (95% CI: 0.833, 0.857) for nephropathy, 0.754 (95% CI: 0.735, 0.774) for retinopathy, and 0.774 (95% CI: 0.761, 0.787) for CAD. The optimal cutoff values of the eGDR were calculated and reported with sensitivity and specificity from the adjusted ROC analysis (Table 4 and Figure 3).

**TABLE 4:** Areas under the receiver operating characteristic curves for Model 3 in discriminating each vascular complication with the optimal cutoff point for eGDR and its sensitivity and specificity.

Complication	AUC	95% CI	Optimal cutoff for eGDR (mg/kg/min)	Sensitivity	Specificity
Nephropathy	0.845	0.833–0.857	6.38	71.0%	88.3%
Retinopathy	0.754	0.735–0.774	4.36	74.4%	65.0%
CAD	0.774	0.761–0.787	4.03	81.7%	60.6%

Abbreviations: AUC, area under the curve; CAD, coronary artery disease; CI, confidence interval; eGDR, estimated glucose disposal rate.

## 4 Discussion

The present study aimed to explore the relationship between eGDR, an indicator of insulin resistance, and complications in patients with type 2 diabetes (T2D). The results demonstrated that microvascular and macrovascular complications of T2D, including nephropathy, retinopathy, and CAD, were significantly associated with eGDR. The risk of all complications was reduced, ranging from 25.5% to 31.3%, with increased eGDR (per SD), independent of well‐known confounding factors for T2D complications. The inverse relationship between eGDR and complications exhibited a nonlinear pattern in nephropathy and a linear pattern in retinopathy and CAD, highlighting the need for adopting a complication‐specific approach while using eGDR as a clinical marker. For nephropathy, the flattened curve at higher levels of eGDR indicates the need for incorporating eGDR as a clinical indicator only for patients with lower and intermediate levels of eGDR. However, for retinopathy and CAD, eGDR yields a proportional effect and is applicable across all eGDR levels. Furthermore, the subgroup analysis for nephropathy, retinopathy, and CAD yielded no significant interaction between groups based on sex, age, and BMI, indicating a consistent effect of eGDR among diverse subgroups.

The results of this study are consistent with those of other studies that explored the relationship between eGDR and vascular complications in patients with type 1 diabetes (T1D). In a study in the UK, lower eGDRs were associated with a greater incidence of micro‐ and macrovascular complications, except for 6 < eGDR < 7.9 for cardiovascular complications, according to the adjusted model [30]. However, in the present study, the association between the eGDR and cardiovascular complications across all eGDR quartiles, compared to the reference quartile, remained significant after adjustment in both models. In another study, Linn et al. showed that the risk of retinopathy and kidney disease was highest in patients with eGDR < 4 mg/kg/min and eGDR between 4 and 6 mg/kg/min, respectively, while those in the lowest quartile did not show any significant risk for kidney disease after adjusting for confounders [31]. In this study, the highest rates of nephropathy and retinopathy were observed in patients within the lowest quartile, showing an eGDR of ≤ 4.74, which contrasts with the previous study's findings. Additionally, our findings showed a significant association between the eGDR and microvascular complications in both adjusted models across all quartiles in T2D patients. These discrepancies can be attributed to variations in the confounders included in the adjusted model and differences in the study population (T2D vs. T1D patients).

There is an ongoing debate regarding the effectiveness of the eGDR in predicting complications associated with type 2 diabetes (T2D). In line with this study, Peng et al. concluded that renal deterioration, determined by eGFR decline, can be predicted by lower eGDR in T2D patients [32]. The findings of the current study showed that in a greater proportion of patients, nephropathy is significantly associated with lower eGDR. Another study showed a significantly lower eGDR in patients with diabetic retinopathy than in patients without retinopathy (6.61 vs. 7.55) [33], which is consistent with this study that showed the highest rate of retinopathy in patients with an eGDR < 4.74 (first quartile). For microvascular complications in the present study, the OR of having a complication was significantly lower in the quartiles with higher eGDRs than in the first quartile.

The adjusted model showed good predictive value for all complications in the ROC analysis. The optimal cutoff point of the eGDR for nephropathy was 6.38, similar to the eGDR cutoff points reported for adverse renal outcomes in another study for all patients [32]. However, the optimal cutoff points of the eGDR for retinopathy and CAD were 4.36 and 4.03, respectively, which were lower than the cutoff point for nephropathy.

The current study established a significant link between insulin resistance, as measured by eGDR, and the complications of diabetes. There is evidence that T2D patients with microvascular complications face a substantially greater risk of macrovascular complications and cardiovascular‐associated deaths [34, 35], emphasising the necessity of the prediction and risk stratification of patients with T2D for both microvascular and macrovascular complications. A large cohort in Sweden with over one hundred thousand participants with T2D revealed that patients with an eGDR higher than 8 mg/kg/min had a significantly reduced risk of stroke and mortality compared to the reference group (36). Similarly, the current study showed that individuals in the highest quartile with an eGDR > 8.38 had the lowest adjusted ORs, ranging from 0.347 to 0.511 for microvascular and macrovascular complications in patients with T2D. Furthermore, our subgroup analysis revealed that the association between eGDR and T2D complications is particularly pronounced in females and individuals aged 65 years and above, in line with findings from Hashemi et al., which emphasised the high prevalence of T2D complications in older adults [36].

The findings of this study suggest that the eGDR, an indicator of insulin resistance, can be used in routine clinical practice as a marker for stratifying the risk of developing complications in patients with T2D and may be helpful in managing and monitoring patients. The eGDR is relatively more affordable than invasive techniques for measuring insulin resistance. Given its low expense and reliance on components routinely evaluated during T2D patients' check‐ups, this offers a significant advantage for both patients and healthcare systems. Additionally, its accessibility for patients increases compliance and reduces any potential stress or discomfort associated with its implementation.

The strengths of the current study include the substantial sample size of individuals with T2D with considerable complication rates, the ability to investigate the association of the eGDR with both micro and macrovascular complications, and the availability of large clinical and paraclinical parameters allowing for adjustment for well‐known confounders for T2D complications. However, this study had several limitations. The study's cross‐sectional design only indicates associations and does not allow for conclusions about causality. Second, the eGDR is a proxy for insulin resistance and may not be as precise as the measurement of insulin resistance by the euglycemic hyperinsulinemic clamp technique. Although a significant correlation has been previously found between the eGDR and clamp technique in T2D patients [13, 37], further studies are needed to validate eGDR against the clamp technique in diverse populations, ensuring its applicability across varying demographic and clinical settings. However, this can also be an advantage since applying the clamp technique for studies with large populations may not be practical. Third, the study population was from a single‐centre diabetes clinic, and the results may not apply to the general diabetes population. Future research using multi‐centre cohorts is warranted to confirm these findings. Fourth, the focus of this study was limited to coronary artery disease (CAD)‐related complications, excluding other cardiovascular and macrovascular complications such as heart failure and peripheral artery disease (PAD) due to a lack of complete data for these outcomes. Finally, the effect of unadjusted confounders, including medication used by the patients, should not be overlooked. It should be mentioned, however, that the present study accounted for several confounders, and further adjustment may cause overfitting of the adjusted model.

In conclusion, this study demonstrated that among patients with type 2 diabetes, a higher estimated glucose disposal rate (eGDR)—an indicator of insulin resistance—was linked to a reduced risk of both microvascular and macrovascular complications. These complications included nephropathy, retinopathy, and coronary artery disease (CAD), and this association remained significant even after accounting for various confounding factors related to type 2 diabetes complications. This suggests that eGDR can be used as a simple, non‐invasive marker in risk assessment and monitoring of patients with T2D. In addition, further longitudinal cohort studies are needed to explore the utility of eGDR in the risk assessment of patients with T2D and compare this marker with established markers for monitoring T2D patients.

## Author Contributions

All authors had a substantial role in management, design, and/or data collection, and/or analysis and interpretation of data, and editing the final draft. Ehsan Bahrami Hezaveh, Rana Hashemi, and Mohammadamin Noorafrooz wrote, reviewed, and designed the study and did the final revision and submission. Sahar Karimpour Reyhan and Amirhossein Yadegar interpreted the patient's data. Ehsan Bahrami Hezaveh, Rana Hashemi, Amirhossein Yadegar, and Fatemeh Mohammadi performed data analysis and designed the figure. Soghra Rabizadeh, Manouchehr Nakhjavani, and Alireza Esteghamati managed and supervised all the authors with their experience and vision. All authors read and approved the final manuscript.

## Ethics Statement

Before enrollment, a specialised nurse took written informed consent from all patients. The ethics committee of Tehran University of Medical Sciences approved the study protocol. The study complied with the principles of the Declaration of Helsinki.

## Consent

All authors have thoroughly reviewed the submitted work and provided their definitive consent for its submission.

## Conflicts of Interest

The authors declare no conflicts of interest.

## Supporting information


Data S1.

## Data Availability

The datasets used and analysed during the current study are available from Dr. Rabizadeh (corresponding author) on reasonable request.
