# Supplementary material for: Estimated Glucose Disposal Rate: A Potential Determinant for Microvascular and Macrovascular Complications in Type 2 Diabetes
Source: Endocrinol Diabetes Metab. 2025 Mar 23;8(2):e70037. doi: 10.1002/edm2.70037 (PMC11931081; doi:10.1002/edm2.70037)
Supplement: Supplementary file 1 — Data S1. [file EDM2-8-e70037-s001.docx]

**Supplementary Material:**

Figure S1: Adjusted logistic regression analysis for Model 3 with ORs and 95% CIs for association of eGDR with vascular complications (color online only)


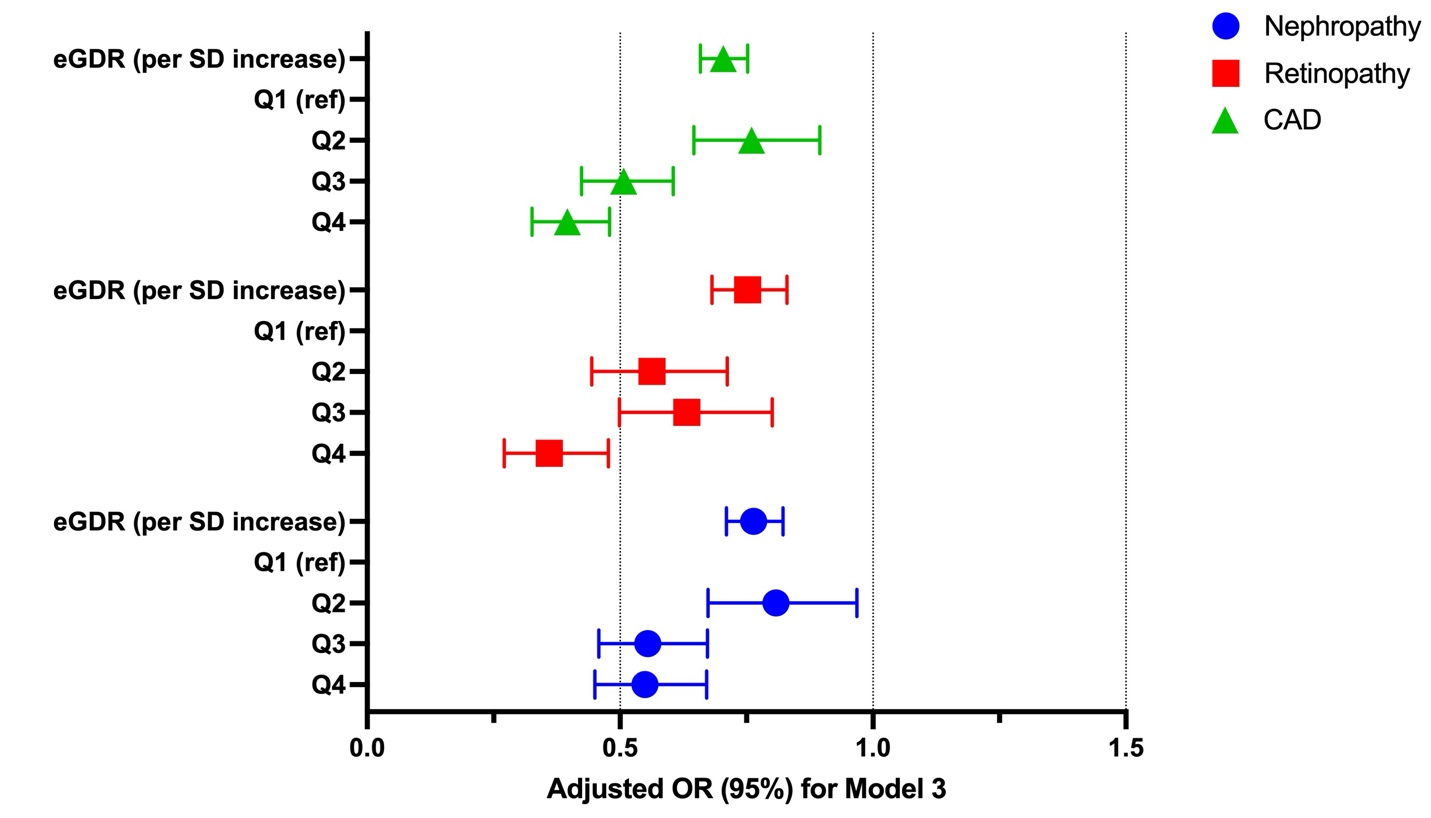


Table S1. Subgroup analysis for association between eGDR and nephropathy, retinopathy, and CAD.

| Complication | Variable | OR (95% CI) | P-value | P for interaction |
| --- | --- | --- | --- | --- |
| Nephropathy | Sex  Male  Female | 0.876 (0.794, 0.967)  0.855 (0.779, 0.938) | 0.009  <0.001 | 0.745 |
|  | Age  < 65 years  ≥ 65 years | 0.873 (0.804, 0.948)  0.831(0.733, 0.941) | 0.001  0.003 | 0.757 |
|  | BMI (kg/mg^2^)  Lean (<25)  Overweight/Obese (≥ 25) | 0.894 (0.765, 1.044)  0.932 (0.865, 1.003) | 0.159  0.063 | 0.294 |
| Retinopathy | Sex  Male  Female | 0.937 (0.810, 1.086)  0.868 (0.777, 0.971) | 0.388  0.013 | 0.527 |
|  | Age  < 65 years  ≥ 65 years | 0.938 (0.836, 1.052)  0.828 (0.715, 0.959) | 0.273  0.012 | 0.194 |
|  | BMI (kg/mg^2^)  Lean (<25)  Overweight/Obese (≥ 25) | 0.871 (0.716, 1.062)  0.913 (0.827, 1.009) | 0.170  0.074 | 0.682 |
| CAD | Sex  Male  Female | 0.756 (0.688, 0.830)  0.817 (0.742, 0.899) | <0.001  <0.001 | 0.403 |
|  | Age  < 65 years  ≥ 65 years | 0.744 (0.684, 0.809)  0.837 (0.752, 0.930) | <0.001  <0.001 | 0.122 |
|  | BMI (kg/mg^2^)  Lean (<25)  Overweight/Obese (≥ 25) | 0.856 (0.743, 0.986)  0.767 (0.712, 0.826) | 0.03  <0.001 | 0.145 |

BMI, body mass index; CAD, coronary artery disease. Adjusted for age, sex, duration of diabetes, BMI, smoking status, eGFR, HDL-C, LDL-C, and TG
